# Injectable facial fillers: imaging features, complications, and diagnostic pitfalls at MRI and PET CT

**DOI:** 10.1007/s13244-017-0575-0

**Published:** 2017-10-04

**Authors:** Pravin Mundada, Romain Kohler, Sana Boudabbous, Laurence Toutous Trellu, Alexandra Platon, Minerva Becker

**Affiliations:** 1 Division of Radiology, Department of Imaging and Medical Informatics, Geneva University Hospital, University of Geneva, Rue Gabrielle Perret Gentil 4, 1211 Geneva 14, Switzerland; 2 Present Address: Division of Diagnostic and Interventional Radiology, Sion Hospital, Avenue du Grand-Champsec 80, 1951 Sion, Switzerland; 3 Division of Dermatology and Venerology, Department of Medical Specialties, Geneva University Hospital, University of Geneva, Rue Gabrielle Perret Gentil 4, 1211 Geneva 14, Switzerland

**Keywords:** Injectable facial fillers, Hyaluronic acid, Silicone, MRI, PET-CT, Granuloma

## Abstract

**Abstract:**

Injectable fillers are widely used for facial rejuvenation, correction of disabling volumetric fat loss in HIV-associated facial lipoatrophy, Romberg disease, and post-traumatic facial disfiguring. The purpose of this article is to acquaint the reader with the anatomy of facial fat compartments, as well as with the properties and key imaging features of commonly used facial fillers, filler-related complications, interpretation pitfalls, and dermatologic conditions mimicking filler-related complications. The distribution of facial fillers is characteristic and depends on the anatomy of the superficial fat compartments. Silicone has signature MRI features, calcium hydroxyapatite has characteristic calcifications, whereas other injectable fillers have overlapping imaging features. Most fillers (hyaluronic acid, collagen, and polyalkylimide–polyacrylamide hydrogels) have signal intensity patterns compatible with high water content. On PET-CT, most fillers show physiologic high FDG uptake, which should not be confounded with pathology. Abscess, cellulitis, non-inflammatory nodules, and foreign body granulomas are the most common filler-related complications, and imaging can help in the differential diagnosis. Diffusion weighted imaging helps in detecting a malignant lesion masked by injected facial fillers. Awareness of imaging features of facial fillers and their complications helps to avoid misinterpretation of MRI, and PET-CT scans and facilitates therapeutic decisions in unclear clinical cases.

***Key points*:**

• *Facial fillers are common incidental findings on MRI and PET-CT scans.*

• *They have a characteristic appearance and typical anatomic distribution*

• *Although considered as safe, facial filler injections are associated with several complications*

• *As they may mask malignancy, knowledge of typical imaging features is mandatory.*

• *MRI is a problem-solving tool for unclear cases.*

## Introduction

"We are all of us stars, and we deserve to twinkle". Marilyn Monroe

The number of people undergoing facial filler injections to get a youthful twinkle has strikingly increased in the last decade. In 2015, more than two million individuals in the USA alone underwent hyaluronic acid (HA) injections of which the majority was for facial rejuvenation [1]. Although middle-aged women still constitute the majority, it is not uncommon to see young adults and older people undergo facial filler injections [2]. These injections, touted as safe and simple “lunch-time procedures,” have become an attractive alternative to incision-needing cosmetic surgery such as facelift procedures. Apart from the desire to look young, other indications include the correction of disabling volumetric soft tissue loss in HIV-associated facial lipoatrophy (HIV-LA), Romberg disease, and post-surgical and post-traumatic facial disfiguring [3–5].

The injection of dermal fillers gives desirable cosmetic outcome by erasing skin rhytides or restoring facial volume loss, or both. The commonly injected sites on the face include the perioral area, periocular region, nasolabial folds, malar fat pad, marionette lines, glabella, and lips. The rising demand for aesthetic procedures has led to the introduction of multiple injectable dermal fillers on the market. The actions by which these fillers produce the desired cosmetic outcome differ from each other and, thus, their complications and imaging features.

A radiologist may be asked to evaluate the complications, extent, and location of a known facial filler injection. The incidentally detected facial filler poses a diagnostic challenge because the patient may forget, deny the history of filler injection, or may not know what type of filler was used [6]. It is a reality of the world market of facial rejuvenation that facial filler injections are not only performed by qualified physicians, but also by many unlicensed practitioners. It is imperative for the radiologist to remain abreast with the commonly used injectable facial fillers, the anatomical context of injection procedures and their complications to avoid misdiagnosis and unnecessary biopsy.

To the best of our knowledge, only very few articles have so far dealt with the imaging features of injectable facial fillers [3, 6–11]. The purpose of this article is to acquaint the reader with the anatomy of facial fat compartments, as well as with the properties and imaging features of commonly used facial fillers, filler-related complications, interpretation pitfalls, and dermatologic conditions mimicking filler-related complications.

## Imaging techniques

Most often injectable facial fillers are detected incidentally on cross-sectional imaging studies. Therefore, radiologists need to be familiar with the imaging features of injectable fillers in order not to confound these with true pathology or vice-versa in order not to miss true pathology obscured by filler injections.

Patients with suspected filler-related complications are most often evaluated clinically and treated accordingly [12]. Therefore, the reported use of cross-sectional imaging in this clinical setting is limited. Nevertheless, dermatologic conditions (cutaneous lymphoma, sarcoidosis, dermatomyositis) may present clinically with non-specific features and imaging can help in differentiating these entities from filler-related complications. The imaging requirements for the evaluation of the facial skin and subcutaneous tissues including facial fillers vary according to the practicing centre. Depending on the availability, radiologists use MRI or high-frequency ultrasound to assess the location and volume of the injected facial fillers and to evaluate filler-related complications. At our institute, MRI is the preferred modality due to its excellent soft tissue discrimination capability, large field of view, and ability to provide anatomic, quantitative, and functional information. MRI has an excellent ability to detect soft tissue inflammation, abscess, and also foreign material in the soft tissues [13]. It is preferred over ultrasound for the localisation of dislodged fillers because it provides anatomical reference [2].

At our institution, the MR examination protocol for facial fillers and dermatologic conditions includes high-resolution thin-slice (512 × 512 matrix for a field of view of 16-20 cm, 1-3 mm slices) acquisitions on a 1.5 or 3 Tesla MRI magnet with surface coils and parallel imaging techniques. We acquire the following sequences: T1 W (axial), T2 W ± fat saturation or a STIR sequence (axial and coronal), diffusion weighted sequences with ADC maps (axial), and post gadolinium injection T1 W with fat saturation. If the injected filler is not known, a “silicone only” sequence with simultaneous water and fat saturation (axial) before gadolinium injection should equally be obtained. A “silicone-only” sequence is designed to suppress all tissues except silicone. It is a combination of inversion recovery–turbo spin echo (IR-TSE) with a TI chosen to suppress fat signal (TI = 230 ms) and a spectrally selective pre-pulse, which suppresses water [14]. Although widely used to image patients with silicone breast implants, the sequence is rarely obtained in other parts of the body. For the detection of injected silicone in the face, the sequence parameters at 3 T MRI that are used in our institution are as follows: TR = 7.8 ms, TE = 3.69 ms, TI = 230 ms, flip angle = 20 deg., voxel size = 1.5 × 1.5 × 6.0 mm, one average, six concatenations. We equally recommend inclusion of a DWI sequence in the protocol for two reasons. First, DWI helps in detecting a malignant lesion masked by injected facial fillers [15]. Second, DWI discriminates between a frank abscess and inflammation and, thus, helps in avoiding futile attempts of percutaneous aspiration [13].

High-frequency ultrasound is a safe, cost-effective, and widely available modality for the evaluation of facial fillers. Various studies have documented its ability to localise commonly used facial fillers. Ultrasound has also been useful to detect filler-related complications, such as abscesses or granulomas localised in the superficial fat spaces. In cases with suspected deep spread of infection, MRI or contrast-enhanced CT (in the emergency setting) is, however, necessary. Operator dependability and poor reproducibility remain major shortcomings of ultrasound in the evaluation of facial fillers [16–19].

CT does not offer advantages over MRI. However, it can identify calcifications, which are a hallmark of certain fillers and filler-related complications (see below). To reduce the radiation exposure, cone beam CT (CBCT) can be used as an alternative to CT to identify calcifications. However, CBCT does not allow assessment of soft tissues. Therefore, contrast-enhanced CT is preferred whenever infectious complications are suspected.

**Fig. 1:**
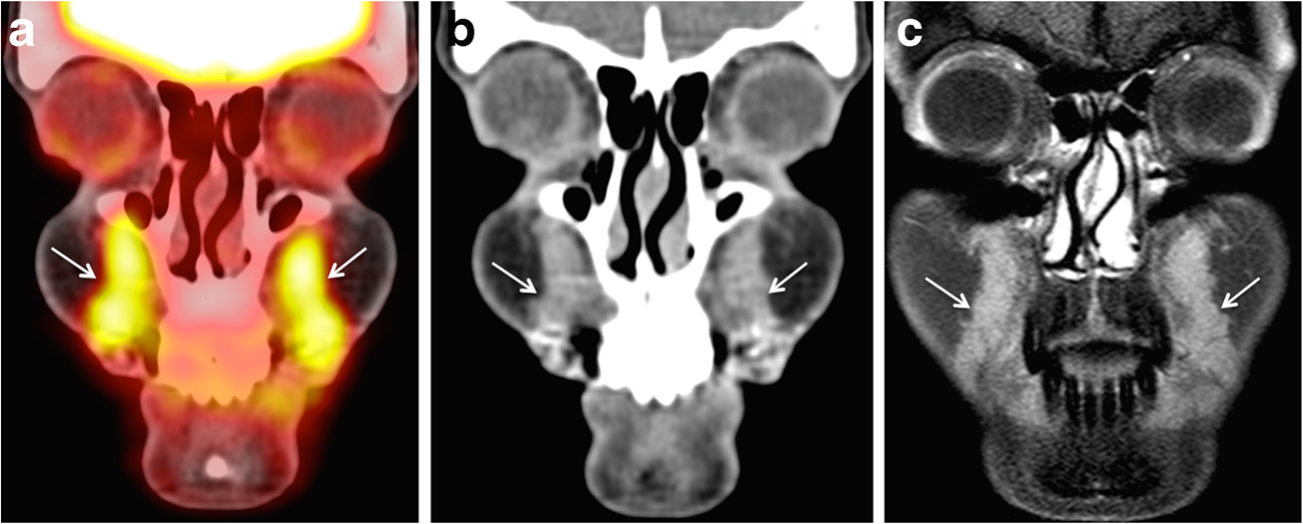
45-year-old female patient evaluated with PET-CT for lymphoma staging. Coronal PET-CT image (**a**) reveals incidentally detected FDG avid areas in bilateral nasolabial fat compartments (arrows). SUVmean = 4.6, SUVmax = 5.9. The areas appear mildly hyperdense on CT (*arrows* in b) and hardly enhancing on post-gadolinium T1 W fat saturated sequences (c, *arrows*). The patient had a history of silicone injections four years earlier. She had no filler-related symptoms

F18- fluorodeoxyglucose (FDG) positron emission tomography-CT (PET-CT) has been found useful for the detection of the source of infection/ inflammation in the body ahead of morphological changes. FDG PET-CT is increasingly used in the evaluation of fever of unknown origin, large vessel vasculitis, complicated sarcoidosis, osteomyelitis, HIV-related infections, and infections in the immunocompromised. However, the use of FDG PET-CT is not recommended for the evaluation of injectable facial fillers as increased FDG uptake is non-specific and can be seen both in patients with and without complications caused by injectable fillers [11]. The increased FDG uptake associated with injectable fillers is a typical pitfall (Fig. 1), which may mimic a malignant tumour or an infectious process depending on the clinical situation.

## Imaging-relevant anatomy of facial fat compartments

Traditionally, surgeons performing facial rejuvenation injections and cosmetic surgeries divided the facial fat into the superficial and deep fat layers separated by the superficial musculoaponeurotic system (SMAS). The SMAS is a three-dimensional fibrous network that connects the periosteum, the muscles of facial expression, the platysma, and the fascia of the parotid gland with the dermis [20]. The superficial fat layer (located superficial to the SMAS) was long considered a single confluent mass, and treatments were designed to lift and reposition the fat pad as one single unit [21]. Similarly, practitioners injected facial fillers in arbitrarily defined facial compartments [21]. More recently, Gosai et al. were the first to classify the superficial facial fat into medial and lateral compartments (units) based on their relationship with the underlying muscles of facial expression on MRI [22]. Rohrich and Pessa demonstrated the actual anatomical divisions of the facial fat by injecting methylene blue dye into cadaveric facial specimen [23]. Subsequent studies by Rohrich et al. and other groups validated the anatomical division of the facial fat and also identified additional distinct compartments [24–28]. In 2012, Girrloff et al. quantified various facial fat compartments on CT scans after injecting iodinated contrast in a cadaveric facial specimen [29].

**Fig. 2:**
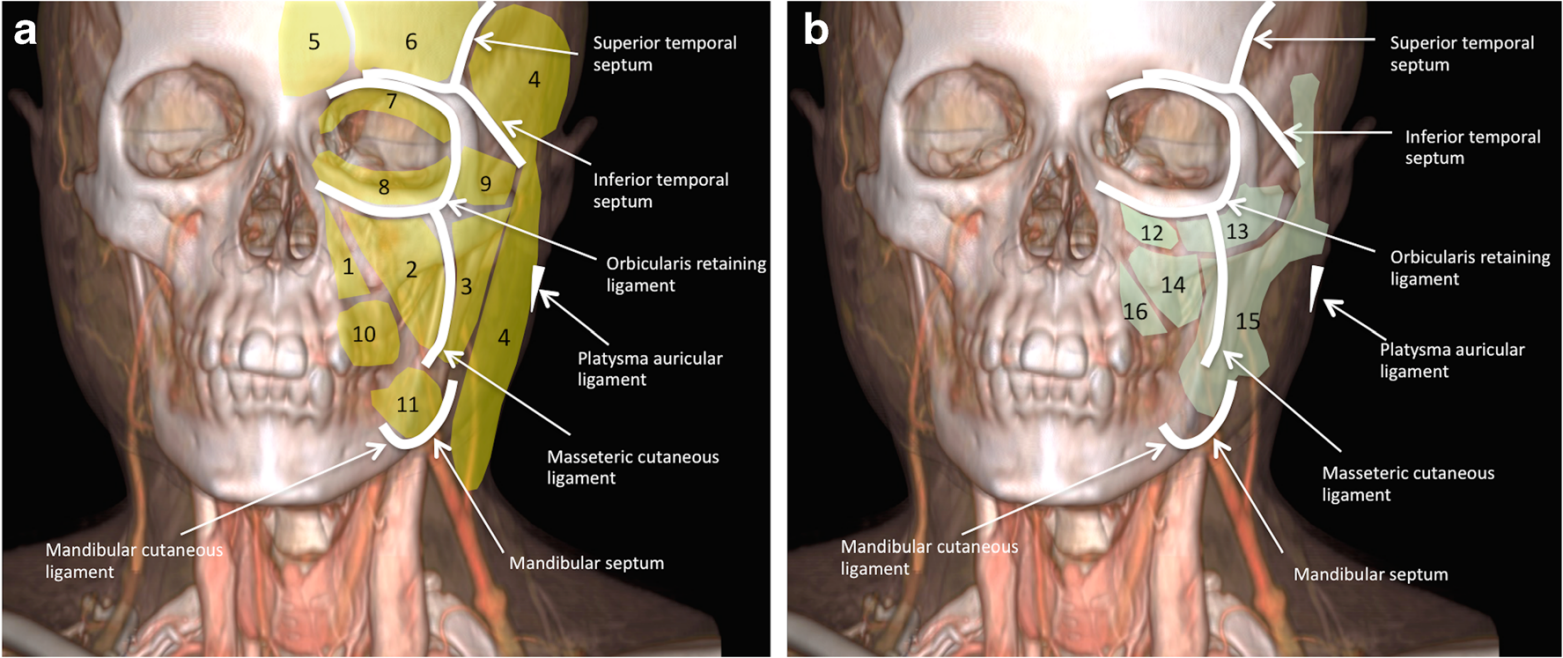
Schematic illustration of the subcutaneous facial fat compartments. 3D reconstruction from contrast enhanced CT. Position of the retaining ligaments of the face (*white lines*), superficial (*yellow*), and deep (*light green*) fat compartments in relationship to the facial skeleton. **a** The superficial group (*yellow*) includes the following compartments (units): the nasolabial fat (1), the medial superficial cheek fat (2), the middle superficial cheek fat (3), the lateral temporal cheek fat (4), the central forehead fat (5) and paramedian forehead (6) fat, the superior orbital (7), inferior orbital (8) and lateral orbital (9) fat, and the superior jowl (10) and inferior jowl (11) fat. **b** The deep group (*light green*) includes the following compartments: the medial (12) and lateral (13) sub-orbicularis oculi fat, the deep medial cheek fat (14), the buccal fat pad (15) and Ristov’s space (16). Modified after Rohrich et al. and Alghoul et al. [23, 83]

**Fig. 3:**
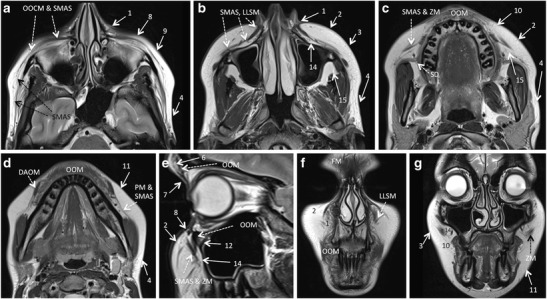
Facial fat compartments as depicted by routine MRI. **a** – **d**. Axial T2 W slices. **e**. Sagittal T2 W image through the mid-pupillar line. **f** – **g**. Coronal T2 W slices. For easier correlation with the schematic drawing shown in fig. 1, the same numbers were used for the superficial and deep facial fat compartments. Fat compartments: nasolabial fat (1), medial superficial cheek fat (2), middle superficial cheek fat (3), lateral temporal cheek fat (4), paramedian forehead fat (6), superior orbital fat (7), inferior orbital fat (8), lateral orbital fat (9), superior jowl fat (10), inferior jowl fat (11), medial sub-orbicularis oculi fat (12), deep medial cheek fat (14), buccal fat pad (15). Muscles and other relevant structures: depressor anguli oris muscle (DAOM), frontalis muscle (FM), levator labii superioris and levator labii superioris aleque nasi muscles (LLSM), platysma muscle (PM), orbicularis oculi muscle (OOCM), orbicularis oris muscle (OOM), superficial musculoaponeurotic system (SMAS), Stensen’s duct (SD), zygomaticus minor and major muscles (ZM)

The superficial fat compartments (between the dermis and the SMAS) and the deep fat compartments (between the SMAS and the periosteum of the facial bones) are illustrated in Figs. 2 and 3. These fat compartments are separated from each other by fibrous membranes, which carry perforator vessels. Schaverien et al. suggested that the highly organised anatomical arrangement of the facial fat and the associated vasculature are probably related to the embryologic development of the facial musculature [30].

Facial fat in these compartments age differently (hypotrophic versus hypertrophic changes or ptosis). For example, ageing causes hypertrophic ptotic changes in the nasolabial fat and middle cheek fat, and hypotrophic involution in the lateral temporal cheek fat and deep medial cheek fat, respectively. In patients with HIV-LA, substantial volumetric changes of the superficial fat compartments (nasolabial, medial and middle superficial cheek fat) occur, whereas deep facial fat units appear to be less affected [31]. Therefore, qualitative assessment of these compartments plays an important role in facial rejuvenation methods and, in particular, in deciding which fat compartments should be volumised [32, 33]. Knowledge of facial fat compartments may also help in understanding facial filler distribution and migration.

Because of its high soft tissue resolution, MRI is excellently suited to depict the facial fat units. Their relative position with respect to the SMAS, orbit and facial skeleton allows correct identification of individual compartments (Fig. 3). Nevertheless, the tiny fibrous septae that separate these compartments, as well as the supporting ligaments of the face cannot be seen on routine high-resolution MR images obtained with standard coils.

## Types of facial fillers and normal imaging features

The Food and Drug Administration (FDA) of the USA considers injectable facial fillers as medical devices used to improve appearance and without health benefit [34]. The classifications of injectable facial fillers vary according to their properties such as nature of the filler, time interval for its biodegradation, and whether it is composed of one or more materials [35, 36]. The filler can be autologous, biological, or synthetic. Autologous fillers consist of the patient’s own body fat. Biological fillers consist of either collagen of bovine, porcine, or human origin or hyaluronic acid (HA) of bacterial origin. Synthetic fillers include paraffin, silicone, calcium hydroxyapatite (CHA), polymethylmethacrylate (PMMA) microspheres, polyacrylamide hydrogel, hydroxyethyl/ethyl methacrylate, and poly-L-lactic acid (PLLA) [36]. Based on biodegradation features, fillers can be classified as rapidly resorbable (<12 months), slowly resorbable (<24 months), and permanent. Rapidly resorbable fillers include HA, collagen and autologous fat. Slowly resorbable fillers include PLLA, CHA and dextran, whereas permanent fillers include liquid silicone and PMMA [36]. Silicone is the most widely used non-resorbable synthetic substance for medical purposes. Of the three forms of silicone, only the liquid silicone is used as facial filler while the elastomer and gel are used in breast implants [37, 38]. The FDA and the European Community (EC) do not recommend all commercially available products. While some products may be FDA approved, they are not EC approved and vice-versa [39, 40]. Besides, some products have a limited approval, which limits their use to the specified areas in the face.

### Autologous fat fillers

**Fig. 4:**
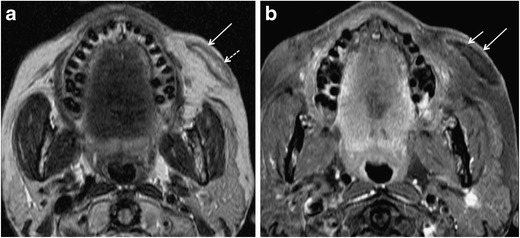
61-year-old woman with discrete facial asymmetry and induration of the left cheek. She had internal fixation of a left tetrapod facial fracture 25 years back. MRI showed a well-circumscribed lesion, isointense to fat on T2 W (**a**) and post-gadolinium T1 W fat saturated (**b**) sequences (*long arrows*). The lesion is located in the left superficial medial cheek fat. Patient confirmed autologous fat filler injection nine months back. The hypointense rim around the filler deposit on T2 and the post-gadolinium rim enhancement on T1 represent a fibrous pseudo-capsule due to scar tissue formation: *dashed short arrow* in (**a)** and *short arrow* in (**b**)

Autologous fat was one of the earliest used injectable fillers to reconstruct facial scars [41]. Its use has declined over the years due to inconsistent resorption rates, which vary from months to years. However, autologous fat is regaining popularity in some parts of the world due to improved harvesting techniques [41–43]. On CT, the filler appears as low attenuation soft tissue. On MRI, it follows fat signal on all sequences and may show a thin pseudocapsule (Fig. 4).

### Collagen fillers

**Fig. 5:**
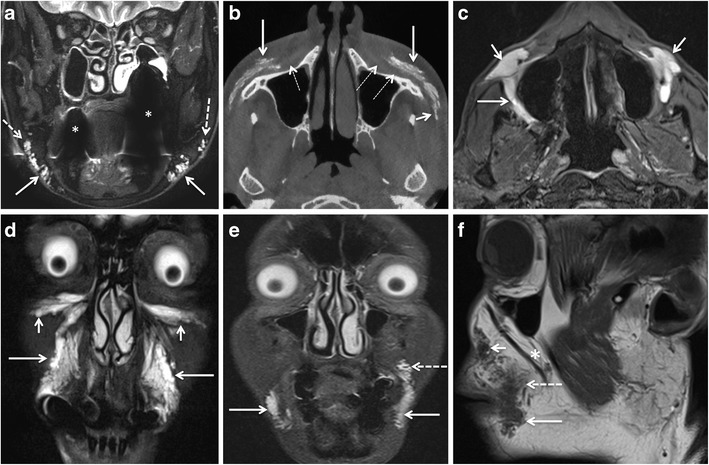
Commonly injected facial fat compartments and characteristic aspect of various fillers at MRI and CBCT. In all presented cases, the patients were symptom-free and were imaged for other reasons. **a** 55-year-old woman with collagen filler injections 5 months previously. On coronal STIR image, collagen filler injections in the superior (*dashed arrows*) and inferior (*arrows*) jowl compartments are seen as hyperintense, lobulated and reticulated areas. Asterisks indicate large artefacts due to dental implants. **b** 62-year-old woman with CHA injections 3 years previously. On axial CBCT with bone window settings, streaky calcific density of CHA filler is seen in the left and right medial and middle superficial cheek compartments (*arrows*) and extending towards the left lateral temporal cheek fat (*short arrow*). Thin dashed arrows point at the SMAS and muscles of facial expression (levator labii superioris, levator labii superioris aleque nasii, and zygomaticus minor muscles). **c** 64-year-old man with HIV-LA and HA injection 6 months earlier. Axial STIR image reveals HA injection performed in the deep medial compartment (*short arrows*) and buccal fat pad (*large arrow*). **d** 45-year-old woman with bilateral HA injections. On coronal STIR image, HA is detected in inferior orbital compartments (*short arrows*) and nasolabial fat compartments (*long arrows*). **e** and **f** 60-year-old woman with bilateral HA injections for cosmetic purposes. On coronal STIR image (**e**), HA injection of the superior (*dashed arrow*) and inferior jowl compartments (*arrow*). Sagittal T1 W image (f) shows hypointense HA in the nasolabial fat (*short arrow*), medial superficial cheek compartment (*dashed arrow*) and jowl compartment (*arrow*). Non-injected buccal fat compartment (*asterisk*)

Collagen is a major structural component of healthy skin. Bovine collagen was the first FDA-approved injectable dermal filler in the USA. The subsequently developed human bio-engineered and porcine collagens gained popularity due to low risk of hypersensitivity reactions as compared to their bovine predecessor. Collagen fillers may last from 6 to 12 months, whereas collagen mixed PMMA microspheres (Artefill) may last up to 5 years [6, 44]. On MRI, collagen appears hypointense on T1 W images and hyperintense on T2 W and STIR images (Fig. 5) due to its high water content [6, 8]. Collagen deposits may show minimal peripheral enhancement in the first 2 months of injection. This minimal enhancement is not indicative of infection. On CT, collagen fillers show fluid attenuation and the injected subcutaneous fat often has a streaky appearance [6]. The imaging appearance of collagen may change if mixed with other substances to prolong its cosmetic effect.

### Calcium hydroxyapatite (CHA) fillers

CHA (Radiesse) comprises spherical microparticles of bone-like composition suspended in an aqueous sodium carboxymethyl cellulose gel. Marionette lines and nasolabial folds have been successfully corrected with CHA. The filler has a tendency to nodule formation and foreign-body reaction, which discourages its use for lip augmentation. The microparticles disintegrate over time, and the volumising effect may last for 1 to 2 years [9, 45]. However, the persisting soft tissue volume gain even after complete filler resorption implies in vivo de novo collagen formation [46, 47].

CHA is hyperattenuating (HU 280-700) on CT and presents with well-defined linear streaks or rounded masses (Fig. 5). CT filler density diminishes after 12 months as the microspheres get absorbed, and eventually the filler may disappear after 24 months [9, 46]. On MRI, CHA fillers have low to intermediate signal intensity on T1 W and T2 W images. They show mild post-contrast enhancement due to vascularisation of the calcified matrix [9, 48]. On FDG PET-CT, CHA appears strongly FDG-avid, which, if detected incidentally, warrants correlation with patient history and morphological imaging to avoid misdiagnosis [9].

### Hyaluronic acid (HA) fillers

HA is a naturally occurring polysaccharide in healthy soft tissue, which binds the collagen and elastic fibres to provide intercellular stability. The naturally occurring HA has a very short half-life. Pre-processing and cross-linking prolong the half-life of HA before its use as filler [43, 49]. Depending on cross-linking HA fillers can be divided into cohesive (monophasic) or non-cohesive (biphasic) gels. The cohesive gel disperses diffusely to fill the small gaps between collagen and elastin bundles, whereas the non-cohesive gel deposits in puddles [44, 50]. The injected HA combines with natural HA in the soft tissue, binds water due to its hygroscopic nature and also induces new collagen formation. Thus, a volume is created, which may last for a few months to 1 year [51–53]. HA fillers are the most widely used fillers for their safety, easy reversibility, and minimal side effects.

MRI with T2 W and contrast-enhanced T1 W sequences can accurately assess the volumetric and temporal changes of subdermal HA filler injection [3, 54]. Because of its high water content, HA filler appears strongly hyperintense on T2 W and STIR sequences and hypointense on T1 W sequences. Injected HA typically shows well-defined serpiginous margins at imaging (Fig. 5). Minor post contrast enhancement is seen in the initial 6 months of injection, which represents increased vascularisation of injected tissue. This minor enhancement and the signal intensity on T2 W images gradually decrease during the first year after injection [3]. The volume gain after HA filler injection is maximal in the first month and remains more or less stable for the next 12 months. Using 3D fat-saturated T2 W sequences, HA was found in anatomical regions situated much deeper than the compartment of the initial injection [3]. The hydrophilic nature of HA and the diffusion permeability of the fibrous septae between the facial fat compartments are thought to be responsible for this finding [3]. As HA binds water in vivo and as the filler also induces in vivo procollagen formation (which has high water content), MRI actually depicts a mixture of all three substances (injected HA, bound water, and de novo formed procollagen) and differentiation between these three components is not possible with MRI [3]. On CT, HA fillers appear as areas of soft tissue attenuation. On PET-CT, they are occasionally FDG-avid [55].

### Poly-l-lactic acid (PLLA)

PLLA (Sculptra), a biodegradable synthetic polymer suspended in sodium carboxymethylcellulose and mannitol has been used for the treatment of HIV-LA and the correction of rhytides [56]. It induces subclinical inflammation with collagen formation and fibrosis [57]. It has a gradual onset of action and results last for a few years [56]. PLLA appears hypointense on T2 W images and shows soft tissue attenuation on CT [6, 10, 47]. On FDG PET CT, there is increased uptake due to the filler-induced subclinical inflammation [58].

### Polyalkylimide and polyacrylamide hydrogels (PAAG)

PAAG (Bio-Alcamid = polyalkylimide and Aquamid = polyacrylamide) are non-biodegradable injectable hydrogel polymers. Aquamid comprises 2.5-4.5% crosslinked polyacrylamide hydrogel and 97.5-95.5% purified water [35, 59]. PAAG induces accumulation of fibroblasts and macrophages, and formation of a fibrous capsule [35]. Correcting nasolabial fold, facial contouring, rhytides, and facial lipoatrophy by PAAG injection is approved in many countries. Delayed reactions like infection, granuloma and migration have been reported [60]. All abscesses reported in a study occurred after polyalkylimide gel [2]. In analogy to other fillers containing large amounts of water, PAAG fillers appear hyperintense on T2 W and hypointense on T1 W sequences [2, 61] and reveal no post-contrast enhancement. On CT, PAAG appears as a well-defined area of fluid attenuation.

### Silicone oil filler

Silicone is a permanent filler, which restores volume and induces new collagen formation. The pure silicone oil is considered inert, minimally antigenic, non-carcinogenic, and a poor medium for bacterial growth [62]. Its use as a tissue filler became controversial due to the reported high rate of complications. Many authors attribute the silicone injection-related complications to the poor injection technique, use of industrial silicone and large volume injections [62, 63]. The off-label use of FDA/CE approved silicone products as facial fillers with microdroplet techniques is reported to have minimal side effects [62, 63]. Illicit silicone oil, however, continues to be in use as facial filler in many parts of the world despite complications.

**Fig. 6:**
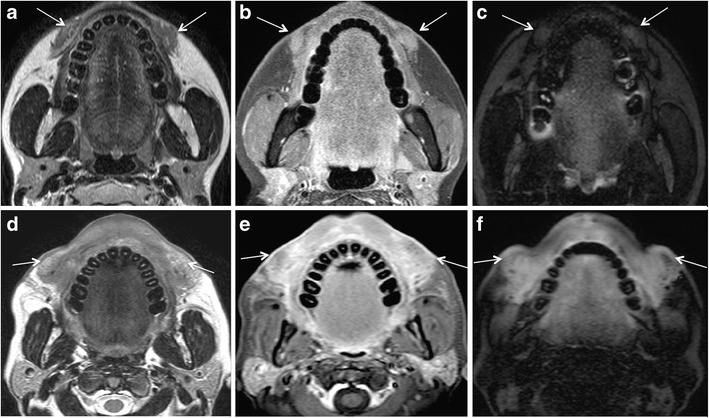
Two different patients with filler injections performed under unclear circumstances (patient 1, 53-year-old woman, **a-c**; patient 2, 46-year-old woman, **d-f**) developed diffuse swelling and induration of the lips and cheeks 1 year after the respective procedures. The injected filler was a mixture of CHA and collagen in patient 1, and it was not known in patient 2. In both patients, the filler and the resulting soft tissue changes show intermediate-to-low signal intensity on T2 W images (*arrows* in a, d), and nodular and strong enhancement on postgadolinium T1 W images (*arrows* in b, e) suggesting an inflammatory reaction. However, in patient 1, the injected areas appear hypointense on the silicone only sequence (*arrows* in c), whereas in patient 2 the injected areas appear strongly hyperintense. Based on these images, the diagnosis of granuloma formation due to CHA and collagen in patient 1 and due to silicone oil in patient 2 was made. Biopsy obtained in both patients confirmed the radiologic diagnosis, in particular biopsy also confirmed the presence of silicone with a characteristic Swiss cheese pattern at histology

The MRI appearance of silicon facial fillers varies according to viscosity and purity. The low viscosity silicone oil is slightly hyperintense to water on T1 W images, iso- or slightly hypointense to water on T2 W images, and hyperintense on the “silicone only” sequence. High viscosity silicone oil is hypointense on T2 W images [6, 10]. A “silicone-only” sequence is designed to suppress all tissues except silicone [14] (Fig. 6). On fat-saturated T1 W images silicone may appear hyperintense and show chemical shift artefact [64]. Post contrast fat-saturated TIW images may show variable enhancement depending on the inflammatory or reactive changes in the surrounding tissues. On CT, silicone appears slightly hyperdense [6]. On ultrasound, it shows a hyperechoic “snowstorm” appearance, which obscures soft tissue details [17–19].

### Autologous fibroblasts

Tissue harvested from the postauricular area is cultured to produce fibroblasts cell lines. These fibroblasts are injected intradermally to correct dermal depressions and rhytides. Autologuous fibroblasts increase thickness and density of dermal collagen. They have a low incidence of hypersensitivity [35, 44].

### Paraffin

Paraffin was perhaps the first substance to be used as facial filler and caused severe granulomatous reactions and “paraffinoma formation” [65]. CT features of “paraffinoma” include calcific rounded foci and soft tissue density nodules with a calcific rim [7].

## Role of imaging for filler detection and characterisation

The detection of injected facial fillers is straightforward with cross-sectional imaging provided radiologists are aware of the typical injection sites (Figs. 1, 2, 3, 4, 5, and 6) and of MRI/CT features. Earlier published studies have demonstrated the ability of MRI in locating all complicated and non-complicated facial fillers in 100% of patients [2, 8, 10]. According to these studies, MRI could detect injected fillers as small as 2 mm in diameter [10], and also those filler-related abscesses and granulomas missed on clinical examination [2].

Regarding filler characterisation, Tal et al. have claimed that they could accurately differentiate between the individual types of injected fillers on the basis of their signature MRI features [8]. The existing literature and experience at our institution do not support their assertions. As discussed in the previous section, most facial fillers (HA, collagen, and PAAG) have a similar appearance on MRI due to their high water content [3, 6, 10]. In our experience, only silicone fillers have a characteristic MRI appearance on the “silicone only” sequence. The “silicone only” sequence is, however, prone to artefacts from non-homogenous fat and water saturation, especially in the presence of dental implants. Therefore, experience and caution are required for its interpretation.

CHA shows characteristic linear or clumps of calcifications on CT [46], whereas all other filler types do not show calcifications unless the injected filler causes foreign body reaction. Foreign body granulomas (FBGs) induced by liquid silicone or paraffin have typical eggshell, rounded, or nodular calcifications [7, 11].

## Complications of facial filler injections and role of imaging

Notwithstanding the much-touted minimally invasive nature of facial filler injection procedures and safety claims of filler manufacturers, all types of injectable facial fillers can cause short-term and long-term complications [38, 40, 66, 67].

**Fig. 7:**
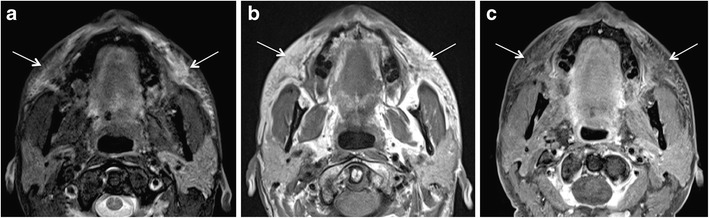
A 49-year-old woman developed bilateral painful facial swellings and redness of skin 4 months after bilateral facial injection of PAAG fillers. On MRI, ill-defined streaky areas of signal abnormalities, hyperintense on axial STIR (*arrows* in a) and iso- to hypointense on axial T1 W images (*arrows* in b), were seen in bilateral superior jowl and medial superficial cheek fat compartments with extension to bilateral medial deep cheek fat and buccal fat pads. These areas display reticulated enhancement on post contrast fat saturated T1 W images (*arrows* in c) suggesting cellulitis/infection. Haematological investigations showed raised inflammatory markers. Patient responded to treatment with antibiotics

Short-term complications are usually related to the procedure itself and early host response to the injected material. Early complications occur within days or weeks of the injection procedure and manifest clinically with erythema, bruising, hyperthermia, swelling, hypersensitivity, nodule formation, and lumpiness in the injection area. They are due to over-injection or mal-distribution of the filler, or they are caused by iatrogenic infection [66, 68]. Infection is rare and has been reported in <0.2% of a series of 1300 patients treated with PAAG; more recent publications suggest an overall infection rate of 0.04% [69, 70]. Infection is mainly caused by inadequate skin disinfection. Infections with *Staphylococcus epidermidis*, *Propionibacterium acnes*, and *nontuberculous mycobacteria* (NTM) usually manifest early or within 3-6 weeks after inoculation. However, delayed manifestations (months to years later) are not exceptional (Fig. 7) [70]. As NTM are known to exist in tap water, infection occurs when tap water contaminates the injection procedure [71].

Occasionally, severe and acute complications like local soft tissue necrosis, blindness, and cerebral infarct may occur due to vascular occlusion [72, 73]. A retrospective study reported acute blindness with glabella and nasolabial fold injection of autologous fat in seven patients, HA in four patients and collagen in one patient, respectively [72].

Long-term complications are related to the injected filler itself and delayed host response. These complications include FBG, delayed manifestations of infection including abscess formation, migration of filler, disfiguring nodules and scarring, tissue necrosis and ulcer, and persistent discoloration [40, 66].

Most complications like erythema, bruising and hypersensitivity do not require radiological evaluation. Non-erythematous nodules formed soon after injection are caused by the uneven distribution of the filler and are likely to resolve spontaneously. Even the nodules caused by inflammation/infection may resolve spontaneously [66]. In one study involving PLLA injection in HIV-LA, patients showed small palpable, painless nodules in 44% cases. Most of these nodules resolved spontaneously [74].

### Abscess formation

**Fig. 8:**
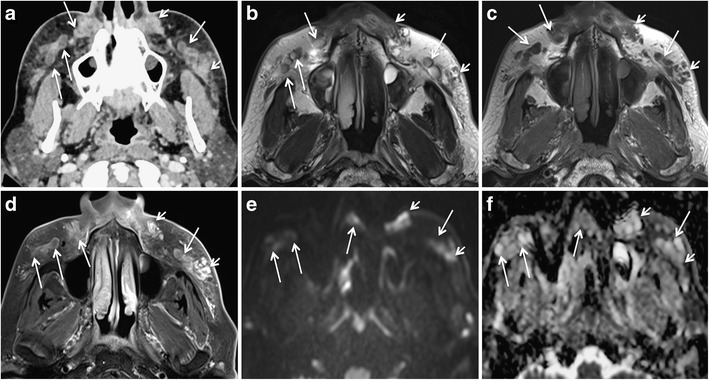
A 65-year-old woman developed pain, erythema, and bilateral cheek swelling after 6 months of HA facial filler injections. **a** Contrast-enhanced CT shows bilateral “grape-like” hypodense, rim-enhancing areas (*long arrows*) and solid appearing enhancing nodules (*short arrows*) in the nasolabial fat, medial and middle superficial cheek fat compartments. T2 W (**b**), T1 W (**c**), and fat-saturated gadolinium enhanced T1 W (**d**) images reveal that the rim-enhancing areas already seen on CT have a high protein content (hypo-isointense on T2 and T1). On b1000 (**e**) and ADC map (**f**), these rim-enhancing lesions show variable diffusivity (long arrows). ADC values were between 1.2 and 2 × 10^−3^ mm^2^/s. The solid lesions (*short arrows*) show strong enhancement on MRI and no restricted diffusivity. Surgery confirmed bilateral infected fluid collections and isolated FBG

The filler injection interrupts the natural barrier of the skin and increases the possibility of infection. Injections performed by untrained hands and use of illicit products increase the risk of abscess formation. Kadouch et al. reported a high incidence of abscess formation with PAAG [2]. Nevertheless, an abscess can occur with all types of filler injections. Like an abscess anywhere else in the body, filler-related abscess appears as a lobulated fluid collection with rim enhancement and adjacent fat stranding on MRI (Fig. 8). On DWI, the abscess may show restricted diffusion [13]. Nevertheless, infected filler deposits and infected fluid collections may show absent restriction on DWI (Fig. 8). To avoid this pitfall, correlation with symptoms and morphologic MR images is essential.

### Non-inflammatory nodule (NIN) and foreign body granuloma (FBG)

FBG and a NIN differ on imaging and histopathology [75]. Differentiation between the two conditions is important as it influences patient management [10]. A FBG is a non-allergic chronic granulomatous reaction that appears months to years after filler injection and grows very slowly. FBG can be multiple and recurring. On histology, FBG has wide spaces between the foreign body particles, abundant macrophages, fibroblasts and giant cells. It shows finger-like projections in adjacent tissues. Although all types of injectable fillers cause FBG formation, FBG is most often seen after long-standing silicone oil injection (“siliconoma”, especially with nonmedical grade silicone), whereas fillers such as HA have a low FBG incidence. FBG shows three histological types: cystic, lipomatous, and sclerotic. Discussion on the histological FBG types is, however, beyond the scope of this article [39, 40, 76].

NINs are single lumps that usually appear 1 to 2 months after a technically erroneous superficial facial filler injection. They occur more often after injections of non-resorbable than resorbable fillers. In patients with HIV-LA, NINs can be seen years after PLLA injection. NINs are usually evenly sized, appear harder and whiter than granulomas, and remain stable. On histology, they show a dense cluster of foreign material, few macrophages, occasional giant cells, and a fibrous pseudo-capsule [39, 40, 76].

**Fig. 9:**
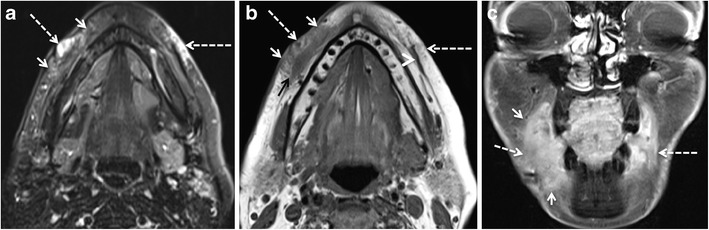
A 57-year-old woman with a remote history of collagen filler injections developed facial lumps. Histologically proven FBGs (*dashed arrows*) in the right and left jowl fat units have high signal intensity on STIR (**a**), low signal intensity on T1 W image (**b**), and strong enhancement on fat saturated T1 W (**c**). The fibrosis around the granuloma (*short white arrows* in a, b) and the thickened right SMAS (*short black arrow* in b) and left SMAS (*arrowhead*) appear iso- hypointense on T1 W and hypointense on STIR images. On coronal post gadolinium fat saturated T1 W image, the degree of enhancement of FBG (*dashed arrows* in c) and of fibrosis around FBG (short arrows in c) appear similar and cannot be differentiated for each other on the basis of enhancement alone

**Fig. 10:**
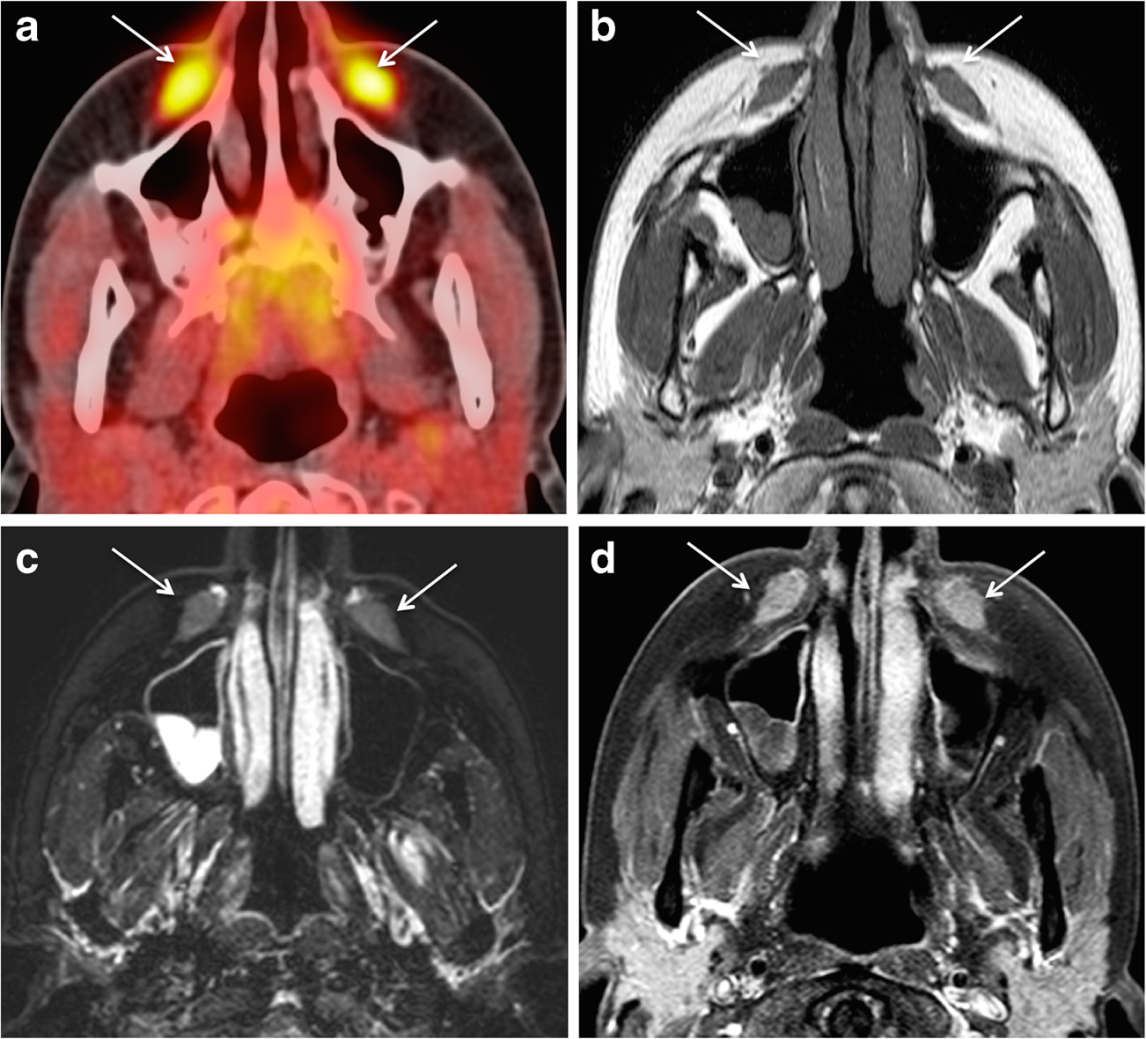
66-year-old woman with PET CT for fever of unknown origin. Palpable indurations of the nasolabial folds. FDG avid areas in bilateral nasolabial folds (*arrows* in **a**) appear of intermediate signal intensity on T2 W (**b**), isointense on T1 W (**c**), and homogenously enhancing on post-gadolinium T1 W fat saturated (**d**) sequences (*arrows*). The location of this signal abnormality prompts the diagnosis of previously injected facial filler. Histopathology showed FBG due to probably PLLA facial filler injections

The MRI features of FBG (Figs. 6, 8, 9, and 10) are described differently in the literature [77, 78]. Girolamo et al. have demonstrated 100% correlation between the post-contrast enhancement around the complicated filler and histological evidence of granuloma formation, whereas non-granulomatous inflammation did not show enhancement [10]. Contrary to this, Tal et al. observed that histologically proven granulomas (*n* = 4) did not show post-contrast enhancement on MRI [8]. According to Kadouch et al., complicated fillers with rim enhancement and adjacent fat stranding (*n* = 11) corresponded to inflammatory nodules on histopathology, and fillers with or without thin rim enhancement were non-inflammatory on histopathology. Kadouch et al. did not dwell on differentiating FBG from non-granulomatous inflammatory changes. In our experience, FBGs show enhancement on MRI; however, the degree of enhancement may vary (Figs. 6, 8, 9, and 10). As suggested by Giorlamo et al., nodular or diffuse enhancement typically suggests FBG, whereas streaky enhancement in the subcutaneous fat corresponds to cellulitis (Fig. 9). On CT, FBGs may show punctate or eggshell classifications [6, 39, 40] and on PET-CT, FBGs show high FDG uptake (Fig. 10).

According to Kadouch et al. [2], clinico-radiologic agreement was substantial for fillers without complications and non-inflammatory nodules (85%), moderate for abscesses (60%), fair for low-grade inflammation (32%), and slight for migration (9%). The patient population in the study of Kadouch et al. comprised patients with filler injections either for cosmetic reasons or treatment of HIV-LA [2]. In our practice, regular use of MRI makes decision-altering contributions to the evaluation of filler-related complications and in patients with both head-neck cancer and fillers. Thus, the utility of MRI for the assessment of filler-related complications might differ in a different set of patients and, also, according to the clinical expertise. Nevertheless, the high cost of MRI warrants its judicious use [10, 79].

According to the literature, FBG is usually treated with intralesional corticosteroid injection, systemic steroid therapy and, occasionally, surgical excision. NIN requires surgical excision and does not respond to intralesional or systemic steroid therapy.

### Migration of fillers and overfilling

Filler migration is a common indication for evaluation with MRI. Poor injection technique has been thought to cause filler migration. Although migration is not typical of any particular filler, permanent fillers (typically silicone) are more likely to migrate due to their longer presence in the body. They may migrate through lymphatic or haematogenous routes and may mimic a malignant pathology of distant organs or granulomatous skin conditions [62, 66, 80]. Authors have showed that MRI was able to detect migrated facial fillers even in the absence of clinical suspicion and denial of a history of filler injection [2, 11].

Overfilling due to excessive filler injection can cause serious patient dissatisfaction. The incidence of overfilling varies from 0.8 to 8% of cases. It may appear as a focal lump or diffuse facial asymmetry. Overfilling due to HA injection can be reversed to some extent by injection of hyaluronidase. Needle aspiration and surgical excision may also be required [11].

### Scarring and lymph node enlargement

Severe chronic inflammatory thickening of the soft tissues may cause significant disfiguring and subsequent scarring. These reactions are common with permanent fillers, especially silicone. CT/MRI may show a thick band-like subcutaneous deposition of silicone associated with diffuse soft tissue swelling and post-contrast enhancement (figure 6) [6, 62, 66]. Mild enlargement of lymph nodes associated with facial fillers is non-specific. The enlarged lymph nodes in case of complicated silicone implants may or may not show silicone contents on imaging [2, 6, 8].

## Pitfalls in image interpretations

**Fig. 11:**
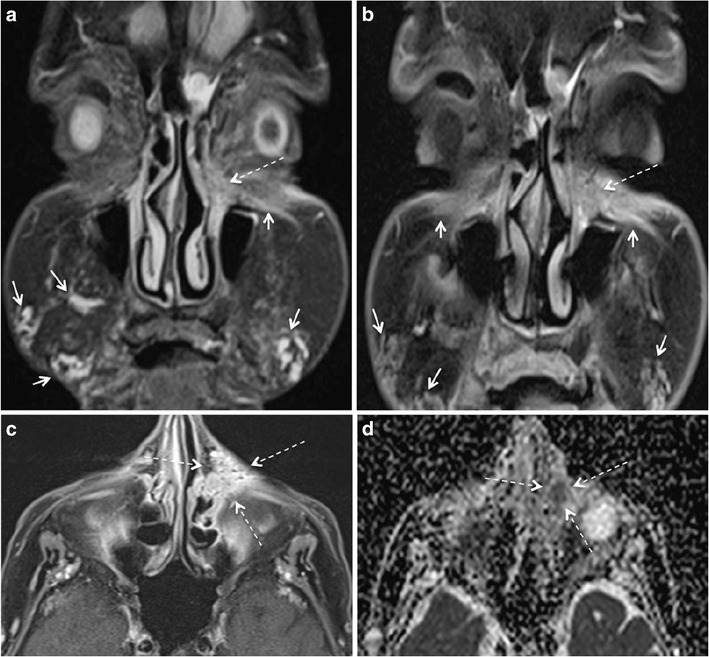
75-year-old woman with unknown facial filler injections eight months back and left epiphora since one month. Coronal STIR (**a**) and coronal gadolinium enhanced fat saturated T1 W (**b**) images show the sites of previous filler injections (*short arrows*) and an irregular mass in the left lacrimal fossa with extension to nasolacrimal duct (*dashed arrows* in a and b). The spiculated, enhancing lesion well seen on the axial fat saturated contrast enhanced T1 W image (*dashed arrows* in **c**). It shows restricted diffusion on the corresponding ADC map (*arrow* in **d**). Furthermore, one would not expect filler injection in this location. Initial histopathology showed evidence of an inflammatory nodule. Repeat biopsy, however, revealed squamous cell carcinoma of the lacrimal sac

Facial fillers may pose a diagnostic dilemma for several reasons. The patient may forget or deny the history of facial filler injection due to social taboo. Injection performed by an unlicensed practitioner may be denied for medical insurance purpose. The patient may not know or remember the type of the injected filler. Incidentally detected or complicated facial filler may mimic recurrent cancer on MRI in patients with previous head and neck cancer. Furthermore, facial filler injection may mask a neoplasm (Fig. 11).

**Fig. 12:**
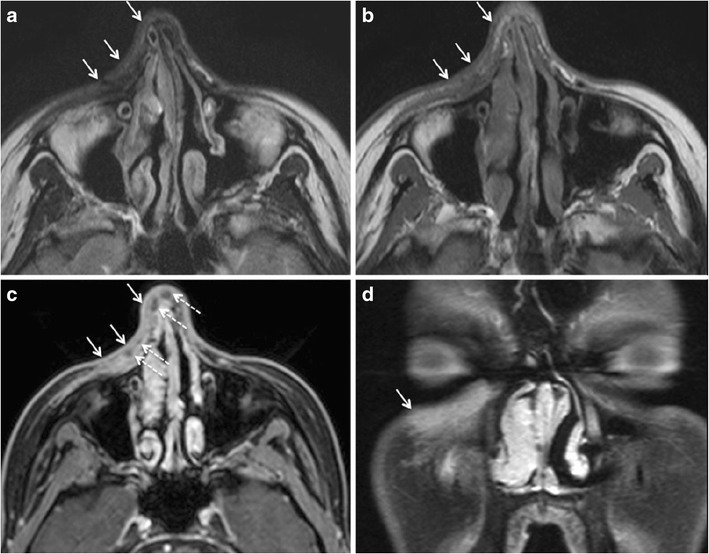
49-year-old woman presenting with slowly progressing subcutaneous induration over the right cheek and with extension to the skin over the nose. Clinically, a facial filler injection related complication was suspected as an underlying pathology. On MRI, the infiltrative lesion appeared hypointense on T2 W (*arrows* in **a**) and on T1 W images (*arrows* in **b**) and showed avid enhancement on post contrast T1 W fat saturated images (*arrows* in **c**, **d**). The lesion involves the superficial and deep layers of the facial fat and the SMAS. Note scattered rounded nonenhancing dark regions (dashed arrows) embedded in the strongly enhancing cutaneous lesion in **c**) possibly suggesting granulomas. The location of this lesion, as depicted by MRI, is not typical for facial filler injection. Based on MRI features, the presumptive diagnosis of sarcoidosis, dermatomyositis, or cutaneous lymphoma was made and biopsy was recommended. Histopathology revealed sarcoidosis. Subsequent CT of the chest (not shown) showed typical interstitial nodules and mediastinal lymphadenopathy. Retrospective analysis of the head and neck MRI revealed no nodal involvement, in particular no “dark lymph node sign” [84]. Nevertheless, the nonenhancing dark regions in **c**) corresponded histologically to sarcoid granulomas, the imaging features being strikingly similar to granulomas in nodes with the “dark lymph node sign”

Lack or denial of history may confuse filler-related complications with dermatological conditions including sarcoidosis, dermatomyositis, and cutaneous lymphoma. On imaging, these dermatological conditions appear mildly hypointense on T2 W images, may show restricted diffusion, and the involved facial soft tissue compartments are atypical for filler injection (Fig. 12). An atypical anatomical location for facial filler injection or migration and restricted diffusion on DWI warrants histopathological correlation to exclude malignancy and other dermatological conditions.

Uncomplicated facial fillers, FBG, non-granulomatous inflammation and abscess associated with facial fillers may show increased uptake on FDG PET-CT/MRI. This uptake is attributed to increased glycolysis in activated inflammatory cells, mainly macrophages [81]. The increased FDG uptake by facial fillers may pose a diagnostic challenge in head and neck cancer and melanoma patients by mimicking a new primary or recurrence, especially if the injection is performed between two follow up scans and the history is denied or simply forgotten. Careful correlation with morphological imaging and the anatomical context of FDG uptake may help in avoiding unnecessary biopsy.

Although FDG CT/MRI is not an imaging modality of choice for the detection of filler-related complications, it is increasingly used for the detection of a source of infection and inflammation, sarcoidosis, and large vessel vasculitis. The increased cost of treating multi-drug resistant infections in immune-compromised patients, diabetics, and elderly patients justifies the use of expensive PET-CT. FDG PET-CT is very sensitive but lacks the specificity to differentiate aseptic inflammation from septic infection [55, 81, 82].

## Conclusion

Injectable fillers are widely used for facial rejuvenation and treatment of post-traumatic disfigurement, HIV-LA and other causes of facial volume loss. This review provides a comprehensive approach to the anatomy of facial fat compartments and summarises key imaging features of commonly used fillers and filler-related complications. Awareness of imaging features of facial fillers and their complications helps to avoid misinterpretation of MRI and PET-CT scans and facilitates therapeutic decisions in unclear clinical cases.

## Abbreviations

CHA: calcium hydroxyapatite
CT: computed tomography
EC: European Community
FBG: foreign body granulomas
FDA: Food and Drug Administration
FDG: F^18^- fluorodeoxy glucose
HA: hyaluronic acid
HIV-LA: HIV-associated facial lipoatropy
MRI: magnetic resonance imaging
NIN: non inflammatory nodule
PAAG: polyalkylimide - polyacrylamide hydrogels
PLLA: poly-L-lactic acid
PMMA: polymethylmethacrylate
PET-CT: positron emission tomography CT
SMAS: superficial musculoaponeurotic system
